# On the edges of extinction: Indigenous whaling governance, the 1977 “bowhead controversy” and its legacy

**DOI:** 10.1017/ext.2024.14

**Published:** 2024-09-09

**Authors:** Sonja Åman

**Affiliations:** Department of Culture Studies and Oriental Languages, University of Oslo, Oslo, Norway

**Keywords:** extinction, environmental governance, conservation, indigenous self-determination

## Abstract

In its nearly 80-year history, the International Whaling Commission (IWC) has shifted from a “whalers club” to an international governance body chiefly focused on the protection and conservation of global cetacean populations. Drawing on recent scholarship on extinction and its entanglements, this article compares two addresses given by whalers at IWC meetings 40 years apart to problematise the way whaling and its relation to extinction is conceptualised in international environmental governance. Guided by practice-oriented document analysis and recent theorisation of extinction as an entangled process, this article analyses the personal stakeholder testimonies from two different representatives of the North Slope whalers of Northern Alaska to the IWC – one in relation to the 1977 Alaska bowhead whaling controversy and the other in the context of the 2018 negotiations over streamlining Aboriginal Subsistence Whaling management and supporting greater flexibility and Indigenous autonomy. By comparing these two statements from very different points of history for the IWC and the governance of Indigenous whaling, this article illustrates some of the ongoing struggles for environmental governance to recognise extinction as a complex, multifaceted process that reverberates throughout human and more-than-human communities.

## Impact statement

This article focuses on the conceptualisation of extinction in the governance of Indigenous whaling practice. This study demonstrates how documents can be used to tell more diverse and multivocal histories about international environmental governance. This article provides historical insight into the internal policy-making procedures at the International Whaling Commission and how colonial legacies of power continue to inform both the drafting and implementation of policies. This article is a useful resource for policymakers, political advocates and other scholars working in the field of Indigenous and environmental governance.

## Introduction

Since the late 1970s, the International Whaling Commission (IWC) has attempted to balance the interests of whalers, anti-whaling advocates and whales themselves in the governance of Indigenous whaling practice. In retrospective, an examination of the first case of Indigenous whaling governance by the IWC reveals the unambiguously unequal, colonial power relations on which much of international governance was built in the 1970s. However, reflecting on the development of the Aboriginal Subsistence Whaling (ASW) framework in the longer term can shed light on the complexities and unexpected outcomes of governing human–whale relationships in the context of a continued threat of extinction.

Extinction – or the threat of extinction – is omnipresent in narratives about whaling. Several conservation campaigns, including the iconic *Save the Whales* campaign, are mobilised by the fear of extinction. Contending with the current environmental crises, research in the environmental humanities and the emerging field of extinction studies emphasise the importance of studying extinction as a process that goes beyond a singular or easily defined ecological event and that is better understood as a complex un- and remaking of relations between the human and the more-than-human. Propelled by the work of scholars such as Van Dooren (2014), Bird (2014), Bird et al. (2017) and Jørgensen (2022), this article discusses the addresses given by Indigenous whalers to the IWC to spotlight how (the threat of) extinction is experienced by communities reliant on whales. By analysing the whalers’ testimonies through an extinction studies lens, this article draws connections between the experiences of whaling communities and the pitfalls of an environmental governance regime focused on the threat of an anticipated future extinction rather than reckoning with the current day reverberations of extinction on communities reliant on human–whale relations.

## Methods and positionality

While customary and Indigenous practises of harvest contribute to a larger scale holistic ontology and ways of being in the world, it is their material reality which ties them to global political and economic structures. For many whaling communities, whales and whaling hold important roles in the processes of social and cultural reproduction (Kalland, 2009; Coté, 2010; Reid, 2015; Durney, 2020, 2024; Sakakibara, 2020). What scientists, politicians and other stakeholders take interest in is the ‘actual’ doing of things – mapping whales, harvesting them and processing and distributing catches. This is why, alongside other qualitative research approaches, examining what documents do is important. Material practises are controlled and regulated through the tools of national and international governance, based on a variety of scientific and policy documentation. In other words, documents and documented records “do not simply describe an external reality ‘out there’: documents also take part in working upon, modifying and transforming that reality” (Asdal, 2015:74).

The IWC has kept comprehensive records of its proceedings since its establishment. Meeting reports, verbatim records and supplementary materials are available through the Commission’s online archive. This article highlights two documented addresses in IWC history, both relating to the customary hunts of bowhead whales (*Balaena mysticetus*) in today’s Northern Alaska. One of these was given in relation to IWC decision-making in the years 1977–1978 and the other in a Commission meeting in 2018. The addresses act as an entry point to a discussion about extinction and the entangled relationship between whaling governance and Indigenous practices and livelihoods (for more, see Viikari, 2022a, 2022b; Åman, 2023). Close reading documents produced by and for the IWC, the analysis of materials here is guided by “practice-oriented document analysis” – a methodological intervention from Asdal and Reinertsen (2021). They argue that following documents offers a critical way to analyse environmental problems as examining “how natural phenomena and environmental problems are brought into and made present in documents (as part of a governmental apparatus, a non-governmental organisation, a scientific community) may come to have a most concrete and decisive significance for these very issues – for example the fate of a species, the management of aquaculture, the extent of oil activities or the regulation of CO_2_ emissions” (Asdal and Reinertsen, 2021:5). Practice-oriented document analysis is used as a guiding framework for working with meeting reports, verbatim records and video recordings. In the discussion section, the two addresses from whalers are brought into conversation with the ongoing work in extinction and extinction studies to illustrate how complicating the concept of extinction could benefit the governance of Indigenous whaling practice, and environmental governance more generally.

As a white^1^ Nordic political ecologist, in my approach, I take inspiration from the idea of “becoming common plantain”, as theorised by Kimmerer (2013) and Stinson et al. (2021). Addressing the challenges stemming from the long traditions of reductive and harmful representations of Indigeneity and the imbalances of power in academic research and writing, the authors advocate for the investigation and deconstruction of the “settler problem” (2021:240). Modelling the behaviour of the common plantain – an invasive plant brought to the Americas by settler colonials but one with many medicinal and practical uses and a limited impact on native ecosystems – they emphasise the capacity of those who identify as settlers to investigate the “settler problem” and to examine and deconstruct structures that support or reproduce colonial power relations. Guided by their instruction, this article focuses on international governance to provide insight into processes that have historically intensified the marginalisation of Indigenous voices and perspectives.

## The IWC

When established in 1946, the IWC consisted mainly of whaling nations. The Commission’s statement of purpose, outlined by the 1946 International Convention for the Regulation of Whaling (ICRW), consisted of a dual aim to “provide for the proper conservation of whale stocks and thus make possible the orderly development of the whaling industry” (ICRW, 1946). Since then, the Commission has grown to a membership of 88 states as of 2024. In the past eight decades, the convention has gone through several modifications. It has been extensively expanded, while the Commission has navigated a rapidly changing global political, economic and environmental landscape. Both the Convention and the Commission have faced criticism from all sides of the political spectrum, ranging from national representatives of whaling nations to environmental activists campaigning for anti-whaling causes (Freeman, 2001; Friedheim, 2001; Creason, 2004; Martello Long, 2004; Wold, 2017).

For the first 30 or so years, the IWC operated mainly as a governance body focused on the regulation of industrial whaling.^2^ The rise of environmental movements and scientific advancements brought many changes for the Commission, making the second half of the 20th century a transformative period for the IWC. The campaign to *Save the Whales*, which gained momentum in the early 1970s, was one of the first globally organised environmental movements to emerge from the growing awareness around pollution, climate change and animal welfare issues (Barstow, 1990; Allen, 2014). As the anti-whaling movement gained momentum and whales emerged as a global icon for environmental conservation campaigns, activists and environmental organisations appealed publicly to the IWC to address the fact that it continued to allow the hunting of several endangered whale species (Epstein, 2008).

In response to the pushback on whaling, the IWC started work on an updated management regime for whaling in the early 1970s. Shifting from a model of industrial resource management more towards marine conservation (Birnie, 1985; Kalland, 1993, 2009; Stone, 2001), the Commission also decided to take on a more active role in the management of Indigenous whaling practices. Records of localised coastal whaling practices date back as early as 6,000 BC, and the variety of hunting techniques range from the use of floats to venomous lances and an array of different harpoon designs (Roman, 2006). Today, the IWC governs five different Indigenous hunts,^3^ but historic and ongoing examples of Indigenous and coastal whaling span the globe from the Arctic to the Caribbean and from Japan to Indonesia. Although the ICRW had always included a passage that specified that “it is forbidden to take or kill grey whales or right whales, *except when the meat and products of such whales are to be used exclusively for local consumption by the aborigines”* (italics added) (ICRW, 1946), the Commission had not intervened with whaling practices of Indigenous communities prior to the changes to the management regime. Increased concerns over the IWC’s conservation and stock protection measures meant that the so-called “aboriginal exemption” also came under scrutiny.

## North Slope whalers and the 1977 “bowhead controversy”

In June 1977, the Scientific Committee (the largest and most influential subcommittee of the IWC) met in preparation for the Commission’s upcoming annual meeting. As per usual, the meeting mainly centred on the management of different whale stocks and sustainable levels of industrial take. Agenda number 13, however, regarded a review status of the so-called “protected stocks”,^4^ and first on the list of things to discuss was the status of bowhead whales. Based on the evaluation of the alarmingly low numbers of bowheads in the Bering and Okhotsk Sea – estimated to be somewhere between 6 and 10% compared to the population size in 1850 – and the recent increase in the numbers of whales struck but lost^5^ by whalers in Alaska, the Scientific Committee recommended a placement of total moratorium on the hunt and catch of bowhead whales. A few weeks later, at the main IWC meeting, the Commission voted 16 to 0 for the removal of “right whales” from the aboriginal exemption, thus banning all and any take (IWC, 1978a).

This relatively brief handling of the matter at the Commission meeting has momentous impact elsewhere. According to the IWC’s species specifications, Arctic bowhead whales are grouped under the larger umbrella of “right whales”. Apart from occasional take by the Siberian Yupik, the Iñupiat and Yupiit whalers^6^ of North Slope Borough in today’s Northern Alaska have remained the only Indigenous communities to hunt bowheads under the exemption. By removing right whales from the aboriginal exemption clause, the Commission effectively terminated the legality of their hunts. The North Slope communities were both upset and disappointed upon receiving the news of the IWC’s decision (Adams, 1982 as cited in Huntington, 1989). In response to the sudden moratorium, nine captains from the whaling villages in North Slope established the Alaska Eskimo Whaling Commission (AEWC). Together, they demanded that the US Commissioner to the IWC to act and launch an official objection to the new amendment (Huntington, 1989).

It is central to note that the North Slope communities themselves had not at any point thus far been heard by the IWC regarding their needs or view on the matter. Although one US representative had suggested that the Scientific Committee leave the matter to be dealt domestically to overcome potentially “complex aboriginal rights issues” (IWC, 1977a:67), little else was done to stop the moratorium from going ahead. The Commissioner did not provide any further context for the North Slope hunts during the 1977 meeting, and in an apparent effort to obtain from publicly objecting to the public commitment pledged by the US political leadership at the meeting,^7^ they were unwilling to raise an objection when the matter was put up to a vote. However, with increased pressure from the AEWC, the Commissioner eventually agreed to bring up the matter at the Special Meeting of the IWC that took place 6 months later in December 1977.

According to the verbatim records of the Special Meeting, after a “considerable discussion”, the Commission accepted a proposal “for a modest take of bowhead whales to satisfy the subsistence and cultural needs” (IWC, 1977d:48) for the North Slope communities. The proposal secured a harvest of 18 strikes or 12 landed whales (IWC, 1977c:4). Considering that during the previous spring season, the number of whales struck was estimated at 106, the suggested quota was notably low. Nonetheless, a reduced quota was arguably better than none for the communities relying on the whaling season. This time, the US Commissioner also voiced their opinion on the proceedings and expressed “deep concern” for way the IWC handled dealing with the “very lives” of Alaskan whalers (IWC, 1977d:60–61). Foreboding some of the more long-standing repercussions of this decision, the Commissioner also pointed out that participants had discussed whether the Scientific Committee was provided adequate guidance on the matter to make such decisions.

Although the original decision was overturned, the sudden six-month moratorium and the reduced quotas left much to discuss at the subsequent annual meeting of the IWC in 1978. The unexpected ban had sparked a public debate in the US^8^ and the federal government was forced to discuss the dichotomy of its own strategy to promote the anti-whaling stance on the international stage, while barely acknowledging the whaling conducted by Indigenous whalers within the country (Huntington, 1989). The North Slope whalers had kept within the previous years’ reduced quotas and even improved their efficiency despite the confusion surrounding the matter. In 1978, in collaboration with the North Slope representatives, the US delegation presented results from their extended bowhead monitoring programme in Alaska. The findings evidenced that the actual population estimates for bowhead whales were higher than the IWC had expected, as the AEWC whaling captains had suspected. After long and strenuous negotiations, the Commission eventually voted to reinstate the Northern Alaska bowhead hunts, although the allocated strike quotas were much smaller than what the villages were used to.

What makes the 1978 meeting one of a kind in the history of the IWC is that it marked the first time a representative of an Indigenous whaling community was given the floor at a Commission meeting. So far, the norm had been for Indigenous whalers to be represented by the government who claimed control over their territories and, at most, observe the meetings as part of national delegations. Thus, the address given by Mr. Edwardsen,^9^ the Director of the Congressional Liaison Office of the North Slope Borough and representative of the Inuit Circumpolar Conference, was a historic first. In the first half, Edwardsen voiced the whalers’ dislike of the IWC’s decision to place bowhead whales under a total moratorium, and he emphasised that after their investment of around 300,000 USD in further research, the bowhead population was found to be bigger than the IWC had estimated (IWC, 1978b:68).

In the second half of his statement, Edwardsen took up what he considered to be a real threat to the bowhead, as well as the North Slope communities. Stating that “(f)rom time immemorial the Eskimos have had a memorable longest experience with the bowhead species longer than any other country at this time” (*IWC, 1978b*:69), he reminded the IWC of the longevity of the shared history between the whalers and the whales. He then called upon the world and the IWC member states to protect the bowhead whales by designating parts of the Arctic waters as a sanctuary, noting that “(t)he Eskimo is not going to exterminate the whale… (t)he jaws of exploitation are in the petrochemical environment” (ibid). Edwardsen closed his speech by addressing environmental activists and other opponents of Indigenous whaling in the audience by requesting that “those people who are for the conservation of the bowhead species, we wish your equal concern for the conservation of the Eskimo as a species”.

Before comparing Edwardsen’s statement with a more recent one given by an AEWC representative in 2018 and discussing some of the contradicting conceptualisations of extinction and human–whale relations in the IWC, it must be noted that the bowhead controversy had a great impact on the governance of Indigenous whaling practice. The events of 1977 led to a re-examination of the IWC’s procedures, and within a few years, the Commission established a separate subcommittee for ASW, which was tasked with redesigning the “aboriginal whaling” clause. Following a series of workshops with natural and social scientists, and some representatives from whaling communities, in 1982, the IWC formalised the term “ASW”. ASW was defined as “whaling for purposes of local aboriginal consumption carried out by or on behalf of aboriginal, Indigenous or native peoples who share strong community, familial, social and cultural ties related to a continuing traditional dependence on whaling and on the use of whales” (IWC, 1982).

For the North Slope communities, the changes taking place in response to their case did not erase the harm caused by the Commission’s actions. This is not to say that the whalers were passive victims – on the contrary, their actions led to a controversy big enough for the IWC to rethink their governance model for Indigenous hunts. However, it did not lead to immediate or sizeable improvement for the whalers themselves. At the end of the controversy and in the years following the implementation of the ASW framework, North Slope villages were left with quotas that totalled mere fractions of their previous catches.

## Looking back and forward: The 2018 address

Although IWC decision-making processes have become more inclusive and integrated more multi-vocality in the past four decades, the impact of the bowhead whale controversy is not neatly contained in the past. North Slope communities continue to whale, and their representatives engage in ongoing advocacy and negotiations with the Commission. During a more recent Commission meeting in 2018, representatives of Indigenous whaling, including the AEWC, testified in support of increased flexibility and security for Indigenous whaling communities.^10^

During negotiations over ASW quota renewals, the current vice-chair of the AEWC, Crawford Patkotak, gave a statement that spoke of the legacy of the 1977 controversy and some of the costs of being governed as a whaler and a member of a whaling community. At the meeting, the IWC negotiated a proposed amendment to the ASW schedule, which would allow an automatic carryover of ASW quotas and thus replace the system in place, which requires whalers to re-apply for their quotas every 6 years (IWC, 2018a, 2018c). In practice, this has meant that every 6 years, whaling communities are faced with the threat of the Commission not approving their hunts. Furthermore, restrictions on carryover quotas put pressure on the hunters to meet their strike limits each season, despite seasonal issues like poor or dangerous hunting conditions.

Patkotak expressed support for the proposed amendment as, for the North Slope villages, it would insure “(their) families are fed nutritiously, (their) elders are cared for, (their) children are educated and that the traditions and practices that bind (their) people together and define (them) continue” (IWC, 2018b). He drew attention to the fact that every few years the villages were required to appeal their case to the IWC and face the possibility that the IWC might deny them their right to whale. He noted that on several occasions, the AEWC had been forewarned that their request might be denied due to “political disagreements” as had happened earlier in 1977 and again in 2002.^11^ He noted that for families in Northern Alaska, such decisions brought “devastating news of (a) threat to (their) food security and cultural survival” (ibid.) Patkotak also reminded the Commission that the 1977 decision had been made against the better knowledge of the whaling captains. He emphasised that the whaling communities remain committed to the cooperative management process currently in place and pleaded for the Commission to “allow our quota to continue based on the health of our whales and our ongoing research, reporting, science-based management and welfare practices” (IWC, 2018b).

Patkotak concluded with reflections on the impact of continued uncertainty has had on them. He listed the considerable amount of work that has gone into adapting hunting methods, improving efficiency and continued training of the whaling captains and crew, on top of the scientific monitoring and liaising with the US government and IWC. He illustrated the extent to which his community has gone to accommodate the demands of the IWC. Regardless of these efforts, Patkotak noted that “despite these many years of working with the IWC and meeting every demand and every challenge in good faith and full compliance, for our people the end of a quota block brings a grave threat to our food security and the life we have lived since time immemorial” (IWC, 2018b).

## Discussion: Complicating extinction in whaling governance

Comparing Patkotak’s address of 2018 to that of Edwardsen’s in 1978 demonstrates the lasting legacy of the bowhead whale controversy and what it represents in the longer history of the IWC. While the knowledge politics and issues of Indigenous self-determination and sovereignty are discussed elsewhere (Epstein, 2008; Kalland, 2009; Roberts, 2010; Viikari, 2022a, 2022b; Åman, 2023), the discussion here draws on the recent work done in theorising extinction to illustrate how the concept of extinction is characterised and negotiated in the discussions about bowhead whaling in these two examples.

Alongside biology and ecology, recent research in the social sciences focused on the multifaceted effects of the current mass extinction event has been brought together under the umbrella of “extinction studies” (Bird et al., 2017). This scholarship centres the complex and diverse processes of loss to better understand and conceptualise the social and cultural impacts of extinction. Van Dooren (2014) illustrates some of the complexities of extinction as a process and unfolds some of the entanglements in the fight against it. Explaining the peril of extinction, he describes how “far more than ‘biodiversity’ - at least in the narrow sense that the term is often used - is at stake in extinction: human and more-than human ways of life, ways of mourning and being with others, even livelihoods and diverse cultural and religious worlds are often drawn into the fray as species move towards, and then beyond, the edge of extinction” (Van Dooren, 2014:7–8). Keeping in mind this expanded definition of extinction, which moves beyond the ecological event of a species lost to consider the reverberating effects of such loss on both human and more-than-human companions beyond the species itself, is helpful for contextualising the stakes of the quota negotiations for Indigenous whalers.

To qualify under the previous “aboriginal exemption” and the current ASW framework, all Indigenous whalers are asked to prove a continuing “dependence on whaling and on the use of whales” (IWC, 1982). With regard to this requirement, it is important to not fall into the trap of perceiving the importance of whaling as tantamount to its importance as a subsistence activity – this idea has been both **a.** proven historically incorrect, as Indigenous whaling practice has long been a commercial activity with a significant role in trade and practices of bordering (see Coté, 2010; Reid, 2015; Shoemaker, 2015), and **b.** been criticised for reproducing the idea of the “tribal slot” by trapping customary practice in a static state of “authenticity” dictated by colonial imaginaries (see Trouillot, 1991; Li, 2000; Muehlmann, 2009; Brigham, 2017; Durney, 2020). For example, at the whalers’ assembly in 2000, a Nuu-chah-nulth chief and hereditary whaling captain, Tom Mexsis Happynook, argued that the ASW framework’s reliance on subsistence is based on “deeply rooted colonial stereotypes of Indigenous peoples as a people perpetually on the edge of starvation, living hand to mouth” (Mexsis Happynook, 2000:4). Rather than reducing whaling solely to a question of subsistence, what the addresses from North Slope representatives highlight instead the multifaceted relationship that whales and whalers share, and how the insecurity around the bowhead whale’s survival and the continuation of this relationship has and continues to reverberate through the communities.

Speaking 40 years apart, both Edwardsen and Patkotak highlight the relationship between the bowhead whale, whaling practice and the cultural and social well-being of the whaling communities. They illustrate how the presence of whales is directly connected to the reproduction of a relationship that has been established since “time immemorial” – one that fosters cultural and food security and enables the passing down of knowledge and traditions. The people of the North Slope, like other Indigenous whalers, are keenly and intimately aware of the radical decline in whales reaching Alaska each year. For the whalers, the threat of bowhead extinction poses the threat of a multidimensional process of loss. Whales and whaling are an integral part of the maintenance of social and cultural practices – a mutually shared way of life. The speakers also attest to the communities’ commitment to walk bowhead whales back from the verge of extinction. Both draw attention to the lengths to which the North Slope villages and the AEWC have gone to improve the efficiency of their practice and to lead the scientific work done for monitoring and protecting populations.

Reading these testimonies in dialogue with extinction studies, they embody the entangled and multifaceted process of extinction in two ways. First, the threat of severing relations that have been fostered across generations – since time immemorial – keenly resembles what anthropologist and scholar of environmental humanities Bird (2014) has called *double death.* She defines double death as “the unmaking of country, unravelling the work of generation upon generation of living beings; cascades of death that curtail the future and unmake the living presence of the past. The death of temporal, fleshy, metabolic relationships across generations and species” (Bird, 2014). The whalers’ statements concretise that the loss stemming from extinction is not only felt in the immediate loss of livelihoods and material practices, but in the loss of continuity and the sense of belonging gained through multigenerational (and ancestral) pasts that have, up until now, been intertwined with bowheads.

Furthermore, the testimonies of North Slope whalers reveal some of the blind spots in conservation and environmental management regimes. Building on Bird Rose and other thinkers in environmental humanities, Jørgensen (2022) discusses historical examples of species extinction and how the threat of future extinction had motivated the work of conservation. Jørgensen notes that extinction is not only in the past (solidified in the moment a species either becomes completely or “functionally extinct”^12^) but also solely looming in the future. Rather, she argues, extinction is present, and it is known through the shared human-more-than-human relations as it is these relations that bridge the temporal bridge between past extinctions and the future threat of them. Jørgensen’s poignant notions of how conservation work is often disproportionately driven by anticipation of future extinction speak to some of the shortcoming experienced by Indigenous whalers.

The IWC and the North Slope whalers work, and have worked, collaboratively to ensure the survival of bowhead whales. Yet, the two are grappling with potential extinction in different ways. The *modus operandi* of the IWC is to suspend the whales’ presence, to avoid an anticipated future extinction. For whaling communities, however, the reverberations of extinction are already present – in the loss of access and the shrinking opportunities to pass down environmental and cultural knowledge that is tied to material, cultural and spiritual practices (i.e., recipes, crafts, rituals) and that maintain their mutual relationship with whales. The significant involvement of communities in conservation work demonstrates the continued centrality of whales, but the statements from Edwardsen and Patkotak convey concerns that maintaining a relationship through these adapted means cannot secure the social and cultural continuity of a relationship that goes beyond them. Their testimonies surpass making their case for a quota renewal as they illustrate how the people in North Slope communities, in many ways, are already living with and adapting to what the IWC considers a (undoubtedly the worst-case) potential future scenario.

Managing the intensifying need for conservation measures without severing relationships and fracturing shared lifeways is a considerable, sometimes nearly insurmountable, challenge for international governance bodies like the IWC. Although the governance of Indigenous whaling has improved significantly in the past four decades when it comes to representational politics and collaboration with Indigenous communities, IWC’s has struggled to frame Indigenous whaling as a practice that is not a threat to whales but one that maintains a relationship and is inherently committed to the survival *and* thriving of bowhead populations. Based on the two addresses discussed here, I argue that frameworks like ASW can benefit from expanding and complicating the concept of extinction and from implementing tools that recognise that extinction sits in complex webs of human and more-than-human relations. Analysing and interpreting (historic and future) testimonies from whalers with a more expansive approach to extinction not only clarifies the gravity of the issue for whaling communities but also allows for a better understanding of the reverberating effects of extinction in the here and now and how to respond to them.

In the wider perspective, how environmental governance regimes define extinction – what it means and for whom – also contributes to the wider dynamics of Indigenous self-determination and sovereignty.^13^ Scholars like Roberts (2010) and Reid (2015) have demonstrated that by defining Indigenous whaling as a “subsistence” activity and prohibiting the whalers’ self-determination over their marine tenure and marine-based livelihoods, conservation and environmental governance have undermined Indigenous communities’ legal and treaty rights. Although the focus of this article is limited to a specific practice, it is paramount that such limited perspectives are not duplicated in the work of environmental governance. Conflicts over whaling and other similar debates, such as hunting and fishing rights, are directly linked to political and legal infrastructures that have historically worked to marginalise and even erase Indigeneity and Indigenous practices (see Harris, 2008; Muehlmann, 2009; Durney, 2020). But they can, too, be used to protect and secure Indigenous futures. Negotiations of extinction and what to do about it when it comes to Indigenous whaling practice must be contextualised in the continuum of loss that many Indigenous peoples and communities have experienced as a part of governance systems more broadly. By taking this legacy seriously and considering measures that might mitigate how extinction is felt and known by communities currently, the IWC has an opportunity to contribute to an era of environmental governance that recognises the challenges of conservation in the Anthropocene and, alongside working against anticipated future extinctions, confronts the realities of living with extinction now.

## Concluding thoughts

Despite the IWC’s undeniably instrumental role in interrupting multiple extinction events through its development and oversight of protective and regulatory measures for industrial whaling in the past eight decades, Indigenous whaling practice has proven to be a challenge for the Commission. Drawing on the literature in extinction studies, this article has analysed two statements from North Slope Borough whalers in relation to the governance of Indigenous whaling practice and, as a result, the millennia-long relationship between North Slope communities and bowhead whales. Through its analysis, this article illustrates that given adequate weight and consideration, testimonies such as these can improve the understanding of how extinctions are experienced in the here and now, and how they already work to modify, disrupt and sever long-standing and mutually held relations. Furthermore, I argue that an expanded and nuanced conceptualisation of extinction could benefit the governance of Indigenous whaling, as reckoning with the reverberating effects (the threat of) extinction is already having on whaling communities can provide pathways for the IWC to develop and integrate improved tools for more holistic governance. In line with established literature linking Indigenous fishing and hunting practices to broader frameworks of sovereignty and self-determination, such tools could potentially include greater involvement and inclusion of Indigenous whalers in leadership and authority positions in environmental management, as well as ways to mitigate the losses caused by extinction.

At the 2018 meeting, after addressing the quota systems for Indigenous whalers discussed in this article, the IWC moved forward with plans to renegotiate aspects of the ASW framework based on recommendations drafted by the ASW SubCommittee and an ad hoc working group on the matter. This process is ongoing. Some of the identified issues with the current system include “removing ASW catch limits from political discussion”, “obtaining adequate information on ASW catch limits and “ensuring local consumption versus commercialism” (IWC, 2014). It remains to be seen whether the renegotiations and resulting amendments will problematise or expand the conceptualisation of extinction, and whether they will make room for greater self-determination for Indigenous whalers to maintain their relationship with whales they have known since time immemorial.

## Footnotes

1 By “white”, I refer to the Nordic feminist and post-colonial conceptualisations of whiteness as a social, political and cultural category and identity, rather than one solely based on race. For more details, see Dankertsen (2019) and Berg (2008).

2 The term “industrial whaling” is used here rather than “commercial whaling” so as to separate non-Indigenous whaling practices from Indigenous whaling practices without erasing the histories of its commercial aspects.

3 These include the hunts by: North Slope Borough communities and the Makah (US), Greenland Inuit communities (Denmark), Yupik and Inuit communities of Chukotka (Russia), and the whalers in Bequia (St Vincent and Grenadines). For more details, see International Whaling Commission (2024).

4 According to the 1975 new management procedure of the IWC, different species were categorised as “sustained management stocks”, “initial management stocks” or “protection stocks” based on their health of the population. Protection stocks included the most vulnerable species and industrial catch was strictly prohibited. For more details, see Gambell (1993).

5 Struck but lost statistics refer to whales that were struck with a harpoon during a hunt but were either not seriously injured and escaped or, if killed, were not retrieved.

6 The communities involved, which include the Iñupiat and Yupiit peoples living in North Slope Borough and its surrounding areas, were referred to as “Alaska Eskimo” in the IWC proceedings. Going forward, they are referred to as the North Slope or Northern Alaska communities or villages, which includes the nine separate villages in the borough that actively participate in whaling and whose hunts were a matter of concern for the IWC. However, to respect the right of self-identification of the whalers, the term “Alaska Eskimo” will be used when it is used by a representative or a member of the communities themselves in the source material, such as in the case of the Alaska Eskimo Whaling Commission (AEWC). Notably, the identifier has recently been emphasised for the shared pride different people mutually hold in subsisting on bowhead whales. For more details, see Sakakibara (2020).

7 The US was open about their anti-whaling stance at the IWC, with the opening remarks from the 1977 Commission meeting including a message from President Carter himself reminding the Commission that “whales have become symbolic of our environmental problems as a whole” (IWC, 1977b:10–11).

8 The AEWC had pressured the federal government into placing an objection to the IWC’s zero quota, but after a few rounds in the District Court, the final court decision sided with the anti-whaling advocates.

9 Mr. Edwardsen’s full name is Charlie Edwardsen Jr., but he is known only by his last name in the IWC records.

10 Due to advancements in recording and archiving methods, observers today can watch the meeting proceedings on video or even choose to follow them as they happen in real time. Audio records of the IWC meetings have been available since 2000 and since 2016, meetings have been recorded on video and uploaded on the Commission’s YouTube channel for public access.

11 Similar to 1977, at the 2002 meeting, the IWC was not able to agree on the matter of renewing quotas for the North Slope whalers, and the villages had to wait with uncertainty until a Special Meeting held later that year to receive confirmation of their quota renewal.

12 Functional extinction is a term used to refer to animal populations that have no possibility of adequately recovering through reproduction.

13 Although not in the scope of this article, there is something to be said about how environmental governance and resource management struggles to see hunting and killing as a legitimate part of these shared ways of life when it comes to whales, although hunting for sport and population control (and even culling) are a regular day occurrence in the management of many other species and ecosystems.
