# Supplementary material for: On the edges of extinction: Indigenous whaling governance, the 1977 “bowhead controversy” and its legacy
Source: Camb Prism Extinct. 2024 Sep 9;2:e10. doi: 10.1017/ext.2024.14 (PMC11895709; doi:10.1017/ext.2024.14)
Supplement: Peer review history

# Editor report: Recommendation: On the edges of extinction: Indigenous whaling governance, the 1977 “bowhead controversy” and its legacy — R0/PR2

**DOI:** 10.1017/ext.2024.14.pr2

**Affiliations:** School of English, University of Leeds, United Kingdom of Great Britain and Northern Ireland

This is a well researched paper, but it requires some revisions, as indicated by the two reviewers. The bulk of the revisions should come in the introductory and concluding sections, where as both reviewers point out more is needed to contextualize the paper as a specific contribution to extinction studies. The assertion that complicating the issue of extinction potentially benefits the governance of Indigenous whaling, in many ways the key argument of the paper, needs particular clarification and reiteration. It would also be useful to point out the further advantages of interdisciplinary approaches to extinction and extinction studies, as this is one of the main subjects of the special issue. Finally, each of the more minor points, mainly of style, that the two reviewers raise need to be addressed.

# Editor report: Decision: On the edges of extinction: Indigenous whaling governance, the 1977 “bowhead controversy” and its legacy — R0/PR3

**DOI:** 10.1017/ext.2024.14.pr3

**Affiliations:** School of Natural Sciences, University of Tasmania, Australia

No accompanying comment.

# Editor report: Recommendation: On the edges of extinction: Indigenous whaling governance, the 1977 “bowhead controversy” and its legacy — R1/PR5

**DOI:** 10.1017/ext.2024.14.pr5

**Affiliations:** School of English, University of Leeds, United Kingdom of Great Britain and Northern Ireland

No accompanying comment.

# Editor report: Decision: On the edges of extinction: Indigenous whaling governance, the 1977 “bowhead controversy” and its legacy — R1/PR6

**DOI:** 10.1017/ext.2024.14.pr6

**Affiliations:** School of Natural Sciences, University of Tasmania, Australia

No accompanying comment.
